# A Case from Practice

**Published:** 1882-06

**Authors:** E. P. Hussey

**Affiliations:** Buffalo, N. Y.


### CASE FROM PRACTICE.

E. P. Hussey, M. D., Buffalo, N. Y.

Was called on the morning of February 13th to see Gracie M., aged three and one-half years; found a severe case of croupous diphtheria: the child was laboring for breath, so that all of the muscles which *could* aid were brought into requisition. The cough was ringing and croupy, and the wheezing, sawing respiration could easily be heard in an adjoining room, and there were all the evidences of constriction and rapidly-increasing obstruction of the larynx. I learned the respiration and cough were of that character all the day before, gradually growing worse until the parents were obliged to hold her in their arms nearly all night. The fauces were highly inflamed, with well-defined diphtheritic patches upon the tonsils; tongue coated white; pulse 136; no desire for nourishment; great thirst; fetor and prostration slight. From the evident course of the disease from the larynx to the fauces, and the character of the cough and respiration, I gave Bromine$^{cc}$, one dose. At eight P. M. found the breathing had been somewhat better through the day but was getting worse again—deposit in the fauces increasing. Gave another dose of the same. In the morning found she had breathed and rested easier all night than during the night before; but she was getting worse again and the membrane upon the tonsils was increasing. As the character of the cough and respiration was the same as at first, I gave a dose of Bromine$^{71m}$, which was followed almost immediately by an amelioration of all the distressing symptoms which continued through the day and night. Upon the morning of the 15th the extensive and deep ulceration of the fauces, and a change in the character of the cough to a hoarser sound, with an accompanying profuse discharge from the nose, induced me to give a dose of Kali Bichromicum$^{cc}$, but as there was no amelioration toward night I gave another dose of Bromine$^{71m}$, which was followed almost immediately by a relief of the subjective symptoms, and from that time the condition of the throat and breathing improved; and I gave nothing more until the morning of the 20th, when I learned she had been unusually talkative all the day before, recalling things which occurred eighteen months previously, and which she would not have been supposed to remember, and that every time she awoke from sleep her symptoms all seemed much worse. I gave her one dose of Lachesis$^{4m}$ at about 8.30 A. M., and before 3 P. M. was sent for in great haste. I found she had had an usually long and quiet sleep, but her mother, becoming alarmed at her almost imperceptible breathing, had awakened her, when she acted so strangely, not being able to speak, and appearing "dazed," that they thought she was dying; however, as she appeared to be rallying somewhat I gave nothing more, and her recovery went on without any further severe symptoms or any more medicine, except one dose of Arsenicum$^{cc}$, on the 23d, given in consideration of the prostration, which then was more marked, accompanied by restlessness at nights. Milk and white of eggs was the only diet, and the local treatment, adjuvants, etc., consisted of a spray of alcohol and water thrown into the throat three times when the deposit upon the fauces was at its height. In self-limited, acute diseases, it is often difficult to say how much the result has been modified by the treatment; but we all know what a case of croupous-diphtheria means, and any such marked arrest of distressing symptoms, following the administration of a remedy, and the favorable termination of such a grave case, must certainly be attributed to the action of the medicine, if anything in the range of experience can be.

